# Clinical analysis of severe visual loss caused by inhalational methanol poisoning in a chronic process with acute onset:a retrospective clinical analysis

**DOI:** 10.1186/s12886-019-1127-9

**Published:** 2019-06-07

**Authors:** Zhonghua Ma, Hanqiu Jiang, Jiawei Wang

**Affiliations:** Department of Neurology, Beijing Tongren Hospital, Capital Medical University, Beijing, 100730 China

**Keywords:** Inhalational methanol poisoning, Visual impairment, Optic nerve, Chronic intoxication

## Abstract

**Background:**

To analyze the clinical features and prognosis of the visual loss resulted from inhalational methanol poisoning in 8 Chinese patients.

**Methods:**

Eight consecutive patients seen at the Beijing Tongren Hospital of Capital Medical University, Beijing, China between January 2003 to August 2017, with complains of vision loss in both eyes, identified as inhalational methanol poisoning. Detailed medical history was extracted. All patients underwent optic nerve and brain magnetic resonance imaging (MRI) scan, laboratory tests, and visual function analysis. Treatment protocols were large dosage of methylprednisolone and B vitamins over 3 months. Patients were seen at 3-month intervals until a year.

**Results:**

Eight patients with optic neuropathy caused by inhalation toxicity of methanol were under observation, whose methanol-contact time spans were form 4 days to 5 years for occupational exposure. All the patients had acute onset, transient systemic symptoms on early stage, both eyes involved with severe visual impairment (visual acuity 0.1 or even worse). Retrobulbar optic nerves (ONs) were the major sites involved. Optic nerve MRI scan showed increased signal of bilateral ONs in the orbit and the canal parts, with enhancement. After treatment, the visual function of these patients got improved in different degree in a year follow-up, but not satisfactorily.

**Conclusions:**

Inhalational methanol toxicity may lead to serious damage to ON in a process of chronic intoxication with acute attack, and with poor prognosis.

## Background

Methanol intoxication is a common problem in many parts of the world. It usually results from either accidental ingestion of methanol or by drinking unbranded and adulterated ethanol. Methanol intoxication is very dangerous because it can cause severe visual dysfunction (including irreversible bilateral blindness), metabolic acidosis, permanent neurological dysfunction and even death [1]. Much less described, is poisoning via non-oral exposure that resulted in severe vision loss. We presented 8 patients who had severe vision loss resulting from inhalation of methanol-containing gas for occupational exposure. In this case series, optic nerve damage confirmed by orbital MRI was the main clinical feature, no typical systemic symptoms of poisoning were identified. Except for common optic neuritis or optic neuropathy, ophthalmologists and neuro-ophthalmologists should attach importance to methanol poisoning by non-oral exposure, in order to manage it promptly and adequately.

## Methods

### Patient selection

This is a retrospective cohort study including 8 consecutive patients seen at the Beijing Tongren Hospital of Capital Medical University, Beijing, China between January 2003 and August 2017. All patients presented to hospital with complains of vision loss in both eyes for inhalation of methanol-contained gases in workshops. All patients were identified as methanol poisoning respectively by two experienced emergency medicine specialists and two neuro-ophthalmologists, and enrolled in our registry. Inclusion criteria were: 1) methanol is primary exposure, 2) intoxication route is inhalation, and 3) no better explanation for the optic nerve involvement. Exclusion criteria were: 1) oral ingestion methanol is confirmed, 2) NMOSD (neuromyelitis optica spectrum disorders), other kinds of optic neuritis or optic neuropathy were confirmed.

### Laboratory tests

Serum and urine samples were collected when these patients were brought to emergency room or outpatient department. Toxicological tests (urine and blood), blood gas analysis, serum anti aquaporin-4 antibody (anti AQP4-IgG), dsDNA antibody, ANA, ENA antibody, CRP, ANCA, and mt-DNA mutation tests were arranged. Intracranial hypertension was excluded by lumbar puncture when it was suspected, including all patients with bilateral optic disk edema. Cerebral spinal fluid analysis was obtained on all patients who agreed to undergo lumbar puncture to exclude infection/inflammation.

### MRI acquisition and analysis

All patients underwent optic nerve MRI performed on a 3.0 T scanner. The MRI protocols included 3 mm thickness T1WI and T2WI sequences with fat suppression, before and after gadolinium administration. The abnormal signals of optic nerve were recorded. Criteria of abnormality: increased signal of ONs on T2WI sequence, with or without enhancement after given contrast on T1WI sequence. All patients also underwent brain MRI to investigate intracranial lesions.

### Visual function analysis

Tests for afferent visual functions were performed before and after treatment. Best corrected visual acuity of both eyes was performed in all patients with international standard visual acuity chart. Visual evoked potentials (VEPs) were recorded with checkerboard pattern stimulation in all 8 patients. Each eye was examined twice in order to check reproducibility of the evoked complex. We evaluated latencies of waves N1, P1, and N2, and amplitudes N1P1 and P1N2. Criteria of abnormality: non-elicitable evoked response, or wave P1 latency above 118 ms, or latency difference between both sides more than 10 ms, or the amplitude is below 3mv. The result of the examination of a patient was categorized as abnormal if at least 1 of the above-mentioned criteria.

### Clinical follow-up and evaluation of response to therapy

All patients were under follow-up prior to the diagnosis given. Therapeutic regime was uniform for all patients: initially with intravenous methylprednisolone (1000 mg for 3 days, 500 mg for 3 days) followed by an oral taper with prednisolone in a starting dose of 1 mg/kg body weight over 3 months. Patients were seen at 3-month intervals until a year. A trained resident doctor kept an additional telephone follow-up of patients, and monitored treatment compliance.

Data was extracted and analyzed by means of descriptive statistics.

Signed informed consents were obtained from all participants.

### Patient and public involvement

There was no patient or public involvement in the design, conduct and analysis of the study. There are no planned dissemination activities towards patients or the general public.

## Results

All patients reported acute, profound, painless, bilateral visual loss. The clinical features of this patient population are presented in Table 1. All patients were young or middle-aged with a mean age of 35.6-year-old, 5 males and 3 females, without any prior significant medical history. 5/8 patients were workers in different small workshops making solid alcohols (the production process:adding methanol into heated stearic acid, and then pouring the mixture into a mold to make solid alcohol) which were widely used in Chinese restaurants; 3/8 patients worked in xylose mill producing xylitol (methanol is one of important raw materials). All of them were informed that they would touch a kind of methanol-containing gas with alcohol-smelling before being employed. Their working places confined and short of ventilation installations. Workers had no protective measures other than work clothes and gloves when they worked. Methanol-contact time of these patients’ spans ranged from 4 days to 5 years. Several days before admission, each patient complained of some systemic symptoms such as fatigue, dizziness, nausea, and difficulty in breathing and so on, followed by disastrous visual loss in both eyes (eye-sight decreased to 0.1 or even worse). Before onset, these patients and their coworkers often suffered from blurred vision, nausea, and dizziness in different degree after working for several hours in workplaces. Once leaving the work environment, the above symptoms could be relieved in a short time.

**Table 1:** Clinical characteristics of patients

Characteristic	Patients (*n* = 8)
Gender	5 male/3female
Mean age in years (range)	35.6 (19–61)
Visual acuity (range) at consultant day	10/16 eyes NLP
	2/16 eyes LP
	3/16 eyes FC
	1/16 eye 0.1
Consulting time after onset to our hospital	13.3 days (3~30 days)
Methanol-contact time spans	13.4 months (4 days~ 5 years)
Visual field defects, *N* = 16eyes	
Isolated central scotoma	16
Neurologic exam, N = 8patients	
Normal	8

Note: *NLP* no light perception, *LP* light perception, *FC* finger counting

**Fig. 1:**
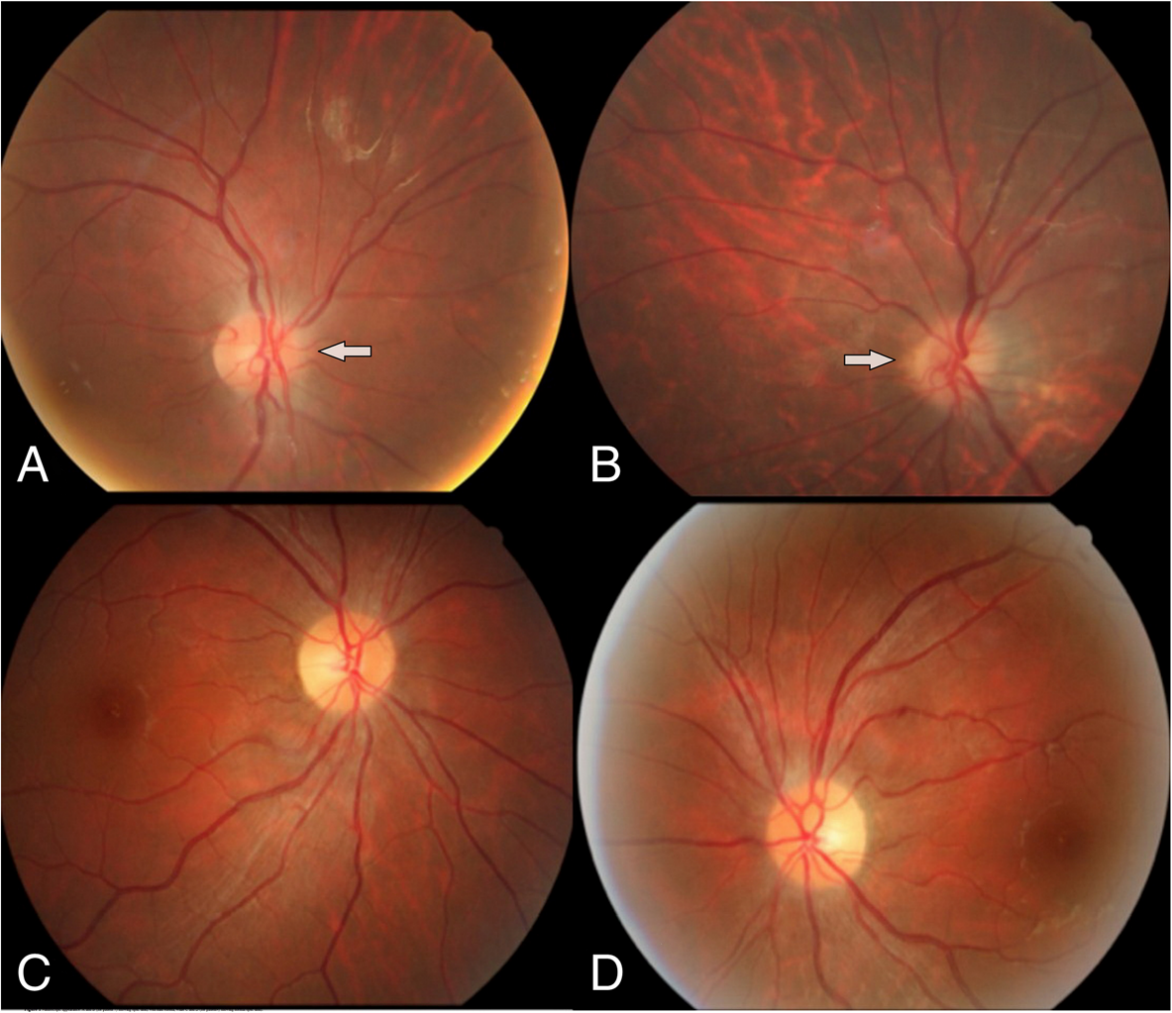
Fundoscopic appearance: **a** and **b** (for patient 7) showing optic disks with mild edema, while **c** and **d** (for patient 4) showing normal optic disks

**Fig. 2:**
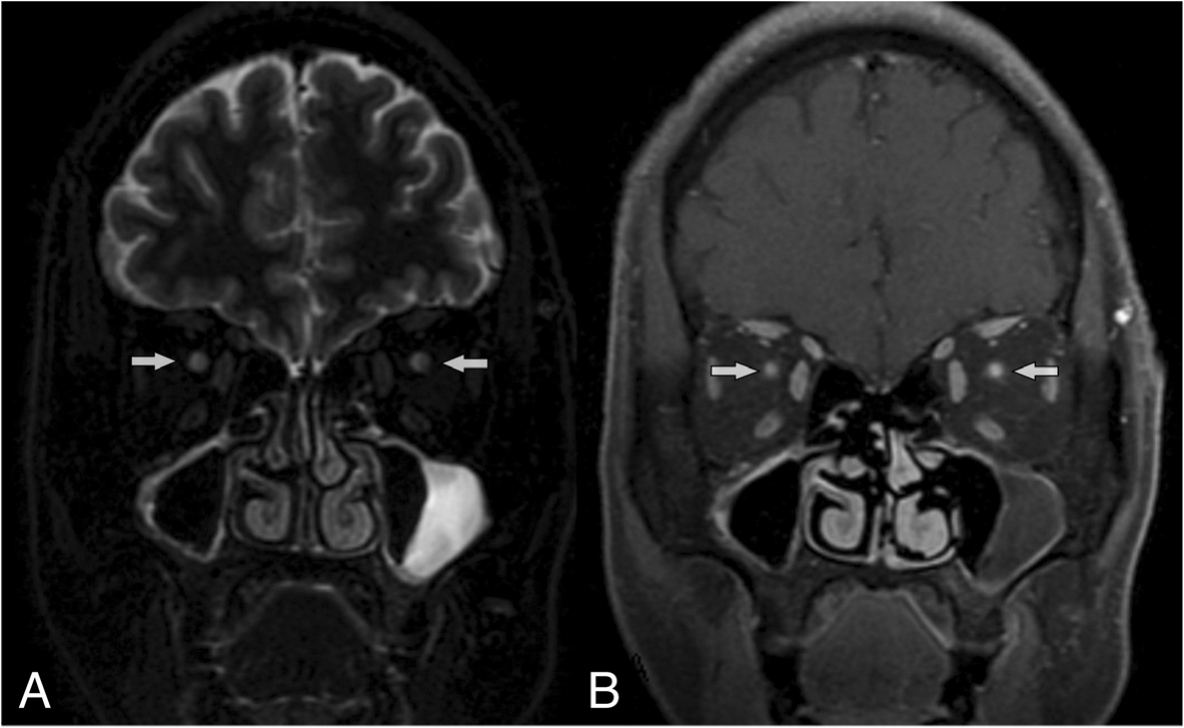
Coronal optic nerve MRI for patient 8. **a** sequence (T2-weighted imaging) showing hypersingnals in both optic nerves. **b** sequence (T1-weighted imaging with contrast and fat suppression) showing enhancement of both optic nerves

Physical examination showed that pupil light reflexes of 16 eyes were abnormal; isolated central scotoma was the characteristic of visual field defects; optic disk edema was found in 6/16 eyes, and normal optic disk in 10/16 eyes (Fig. 1); other neurologic examination results were normal. The results of auxiliary examination were shown in Table 2. Visual evoked potentials (VEPs) showed no fixed waveforms or poor waves in 15/16 eyes. All patients had poor eyesights as showed in Table 1, so they could not hold their eyes in fixed position. Abnormality of VEPs consisted in unelicitable evoked response in 13/16 eyes, and in latency prolongation in 2/16 eyes, which were combined with the decrease in amplitude. The values of prolonged latency were 141 ms and 143 ms separately. Optic nerve MRI with contrast demonstrated increased signals of bilateral optic nerves in the orbit and the canal parts, with enhancement (Fig. 2). Brain MRI showed non-specific demyelination in white mater or normal. Toxicological tests (urine and blood) were negative in all patients. The results of blood gas analysis and serum anti- AQP4-Ab tests were negative. All of the patients were transferred from several different primary hospitals in remote areas. Except one patient who had received hemodialysis, none was treated with ethanol, fomepizole, hemodialysis or methylprednisolone before being transferred. After treatments of large dosage of methylprednisolone combined with B vitamins in our department, the visual function of 5/8 patients got improved in different degree, but not satisfactorily, as shown in Table 3.

**Table 2:** Auxiliary examination details of patients

Examination items	Patients (*n* = 8)
Brain MRI appearance	
Non-specific demyelination in white mater	2 patients
Normal	6 patients
Orbital MRI	
Increased signals of bilateral optic nerves in the orbit and the canal parts, with slight enhancement	8 patients
VEP appearance	Poor waves or without fixed waveforms in 15/16 eyes
Fundoscopy	
Optic disk edema and pale	6/16 eyes in 3 patients
Optic disk normal	10/16 eyes in 5 patients
Toxicological tests (urine and blood)	
Positive	None
Negative	8 patients
Anti- AQP4-Ab and other antibodies	
Positive	None
Negative	6 patients
Undone	2 patients
Blood gas analysis	
Normal	8 patients
Abnormal	None

**Table 3:** Detailed information of treatment regimen and effect

Patient	Exposure time-span	Consulting time after onset (to our hospital)	Treatment regimen	VA (Snellen visual acuity) before treatment	VA After onset for a year
Patient1 M/34y	>2 years	20 days	Methylprednisolone, B vitamins	OD 0.1 OS 50cmFC	OD:0.1 OS:1mFC
Patient2 F/26y	9 months	3 days	Methylprednisolone, B vitamins	OD NLP OS NLP	OD 1mFC OS 0.05
Patient3 M/43y	5 years	13 days	Methylprednisolone, B vitamins IVIG	OD NLP OS NLP	OD NLP OS NLP
Patient4 M/30y	3 months	10 days	Methylprednisolone, B vitamins IVIG	OD NLP OS NLP	OD FC OS HM
Patient5 F/26y	8 months	16 days	Methylprednisolone, B vitamins IVIG	OD NLP OS LP	OD HM OS 0.05
Patient6 M/61y	1 month	7 days	Methylprednisolon, B vitamins	OD NLP OS NLP	OD NLP OS NLP
Patient7 F/19y	2 months	7 days	Methylprednisolone, B vitamins Dialysis (before)	OD LP OS NLP	OD 0.1 OS FC
Patient8 M/46y	4 days	30 days	Methylprednisolone, B vitamins	OD FC OS FC	OD FC OS FC

Note: *OD* Oculus Dexter, the right eye; *OS* Oculus Sinister, the left eye, *FC* finger counting, *LP* light perception, *NLP* no light perception

## Discussion

In current report, we presented 8 patients suffering inhalational methanol poisoning in a chronic course with acute attack. Optic MRI, as a new examination for this disease was performed in this patient group and showed abnormal signals in both ONs of each patient, which served as more affirmation that ON was the vulnerable target.

There have been many reports on ingestion methanol poisoning, but inhalational route, less reproted, is also an important way of exposure. A case report published in 1990 described the “unusual presentation of solvent abuse” in a 17-year-old male who appeared intoxicated after inhaling a rag soaked with carburetor cleaner containing methanol [2]. Another research described seven cases of intentional inhalation abuse of carburetor cleaner. These patients experienced CNS depression, visual disturbance (one case), death (one case), and acidosis (three cases) [3]. A retrospective chart review reported eighty-seven cases had inhalation as the route of exposure with bad prognosis [4]. However, some researchers believe that significant toxicity following inhalation of methanol containing solvents is rare and at low risk for methanol to induce complications of visual dysfunction and refractory acidosis [2, 5]. Almost all these researches focused on intentional inhalation abuse of some methanol-containing solvents, for addiction or suicidal intent. By contrast, occupation-related inhalational intoxication was reported more rarely. Recently, a report from Korea presented 3 patients who had severe neurological symptoms: acute visual loss, mental changes and convulsions, accompanied with metabolic acidosis resulting from non-oral methanol poisoning for the high methanol concentration in the air of workplaces [6].

This report is built upon these observations to provide a kind of much more chronic inhalational methanol intoxication due to occupational factors, with an acute onset. Except one patient in this report, the exposure history of others was more than one month, the longest 5 years. Although we did not get the positive results of toxicological tests of methanol in serum and urine of these workers, clinical evidences including the evidential medical history, typical clinical manifestations and similar occupation-exposure experiences made us give the definite diagnosis of methanol poisoning according to the diagnostic criteria and principles of occupational acute methanol poisoning. We suggest that much rarer transdermal toxication should be considered. Percutaneous exposure of methanol is possible when clothes are contaminated with a large amount of methanol. It is believed in some geographic regions that methanol applied on the skin could treat muscle and joint pain. In several previous reports, patients who had placed the methanol-soaked dressing on the skin to relieve muscle pain developed poisoning symptoms [7–9]. The patients in this paper denied the history of methanol exposure in a similar manner. Moreover, their recurring early symptoms of poisoning could be improved in a short time after leaving the workplaces. So, we believe that respiratory inhalation is the poisoning route for these workers. The negative results of toxicological tests were due to the short half-life period of methanol in body and the delayed consulting time to our hospital. The differential diagnoses, including NMOSD and other optic neuritis or optic neuropathy, were under consideration, but were excluded eventually according to the exposure history and assistant examinations.

These patients in our reports showed different clinical characteristics from ingestion poisoning: (1) Severe visual loss was the main symptom, and might be a chronic poisoning course. As well known, methanol intoxication can cause severe visual dysfunction, especially irreversible bilateral blindness [1]. In current observation, bad visual function was still the outstanding manifestation. But 3 patients complained of constant slight blurred vision in one or both eyes before the acute visual loss, hinting the possibility that optic nerves had been partly injured since several months, which accorded with the characteristics of chronic intoxication. Moreover, the orbit MRI scans provided powerful evidence for ONs damage. Visual evoked potential demonstrated no potential or poor wave forms, which also suggested that the optic nerve damage was serious and bad prognosis. The selective optic nerve toxicity of methanol is striking and quite likely occurs because of a direct effect of the formic acid (metabolite of methanol in the body), which is thought to cause interruption of mitochondrial function in optic nerve, resulting in optic injury and loss of vision [10, 11], which is consistent with studies that showed high cytochrome oxidase activity in human optic nerve [12]. (2) The systemic symptoms were transient and self-limiting, while blood gas analysis was normal, and no distinctive abnormal focus or bleeding were found in basal ganglia regions on brain MRI images. Systemic effects are mainly due to metabolic acidosis resulting from accumulation of formic acid. In contrast to the cases of Korea [6], these clinical features were thought to be related to lower volume of inhaled methanol in a short period and lower concentration accumulated in body. In addition, the short half-life period of methanol and the delayed consulting time to our hospital contributed to these negative results of laboratory tests. However, this kind of lower poisoning still lead to disastrous visual impairment. It is well-documented that the level of toxicity, which does not correlate with the serum methanol level and hence, is not considered a good indicator of prognosis [13]. (3) Optic nerve MRI with contrast showed abnormal signals in optic nerve. To our knowledge, orbital MRI scan is rarely taken to observe the optic nerves in patients with methanol poisoning. Optic nerve and basal ganglia are the tissues most at risk from methanol intoxication for metabolic or apoptotic sensitivity to methanol metabolites. The typical manifestation on brain MRI scans in patients with ingestion poisoning was hemorrhagic or non-hemorrhagic necrosis in the bilateral putamen, occasionally in frontal and insular cortices and subcortices [14, 15]. But manifestation on orbital MRI scan for optic nerve in these patients is rarely reported. In our cases, increased signals of bilateral ONs in the orbit and the canal parts with enhancement were the common features (Fig. 2). All these data confirmed that the retrolaminar regions of the optic nerve were vulnerable to damage in methanol poisoning. However, these findings do not fully represent the characteristics of injured optic nerve in methanol poisoning, for most cases of methanol poisoning are caused by oral ingestion, not by inhalation.

In our report, we did not try the treatment protocols of dialysis or detoxification drugs, for the results of blood gas analysis and toxicology tests were normal. Optic nerve MRI showed abnormal signals with enhancement in all patients, indicating the presence of optic nerve inflammatory reaction. Retrobulbar optic neuritis or neuro-retinitis is the well-reported mode of presentation in cases of acute methanol poisoning [16–18]. The routine treatment regimens include the use of ethanol, fomepizole, folinic acid, sodium bicarbonate, and hemodialysis, etc. [10, 18, 19]. These treatments mainly prevent the formation of formic acid and further toxicity, but do not have any effect on established ocular inflammation [18]. There are some studies reporting the use of intravenous methylprednisolone, resulting in good visual outcomes [11, 18, 19]. In a report, Shukla et al. used intravenous methylprednisolone for 3 days followed by oral prednisolone, which resulted in a significant visual improvement in all of the patients except one [18]. Therefore, all the patients in our observation were treated with intravenous methylprednisolone 1000 mg for 3 days, 500 mg for 3 days, followed by oral prednisolone and B vitamins. The visual function of 10 eyes in 5/8 patients got improved in different degree, as shown in Table 3. But compared to the visual functions before treatment, the improved visual function was not satisfactory, which is inconsistent with the results of foreign studies. And 6 eyes in 3/8 patients had no improvement in visual function after the above treatments. Carefully reviewing the medical history of these three patients, we found that longer methanol-contact time (>5 years), elderly (61-year-old), and delayed treatment (> 1 month) were their respective reasons. Additionally, except for ethnicity effect, we speculated that chronic optic nerve injury caused by chronic methanol-inhaled poisoning was also an important reason for the poor prognosis. The inhalation of methanol to the poisoned dose might be a chronic process of accumulation of methanol and its metabolites in body before acute onset. It should be noted that the prominent visual symptoms may compel patients to consult an ophthalmologist or neuro-ophthalmologist time and again. Without typical intoxication history, after differential diagnosis including NMOSD, various optic neuritis and optic neuropathy were all excluded in outpatients, these patients had missed the best treatment timing. Although proper treatments were given, the severe vision symptoms of these patients did not get well-relieved. However, we believed that the medical treatment might prevent further damage to the optic nerves according to the treatment effect. By comparison, cases of methanol poisoning caused by oral ingestion experienced an acute attack following just once intake or misusing of methanol, with obvious systemic symptoms such as mental changes, extrapyramidal symptoms and convulsions. Most of the patients would typically seek medical attention, and they might be cured or partially improved after given appropriate treatment timely.

## Conclusions

Recently, more and more attention has been paid to inhalation methanol poisoning. These cases in our report presented severe vision loss and transient systemic symptoms resulting from this distinctive intoxication. Methanol poisoning via non-oral exposure can also cause severe neurologic complications especially dramatic loss of vision, and might be in a chronic process. It is suggested that emergency physicians, ophthalmologists and neuro-ophthalmologists should be aware of inhalation methanol poisoning and raise their vigilance against the disease. Accurate and fast diagnosis and effective treatments guarantee the better prognosis.

## Data Availability

The data set available from the corresponding author on reasonable request.

## Abbreviations

AQP4: Aquaporin-4
MRI: Magnetic resonance imaging
NMOSD: Neuromyelitis optica spectrum disorders
ON: Optic nerve
VEP: Visual evoked potential
